# Independent and joint associations of sleep quality and physical activity with glycaemic control in patients with type 2 diabetes mellitus

**DOI:** 10.3389/fnut.2025.1694982

**Published:** 2025-11-03

**Authors:** Lei Zhou, Yize Li, Shuo Zhang, Haimeng Zhang, Xiaomei Ji, Yuanyuan Yu, Hong Xie

**Affiliations:** ^1^School of Public Health, Bengbu Medical University, Bengbu, China; ^2^School of Nursing, Bengbu Medical University, Bengbu, China; ^3^Department of Nephrology, The Second Affiliated Hospital of Bengbu Medical University, Bengbu, China

**Keywords:** T2DM, sleep quality, physical activity, glycaemic control, joint effect

## Abstract

**Background:**

Although determinants of glycaemic control in type 2 diabetes mellitus (T2DM) have been extensively investigated, the joint influence of sleep quality and physical activity (PA) remains insufficiently studied. We aimed to examine the independent and combined associations of sleep quality and PA with glycaemic control in patients with T2DM.

**Methods:**

We conducted a cross-sectional study of 329 patients with T2DM. Sleep quality was evaluated using the Pittsburgh Sleep Quality Index (PSQI). PA was self-reported through questionnaires and expressed as metabolic equivalents (METs). Associations between sleep quality, PA, and glycaemic control were assessed using multivariable logistic regression.

**Results:**

After adjusting for confounding factors, we observed that declining habitual sleep efficiency was associated with an increased risk of suboptimal glycaemic control (OR 1.64, 95% CI 1.14–2.43). Participants with poorer sleep quality had a higher risk of suboptimal glycaemic control compared with those with better sleep quality (OR 2.09, 95% CI 1.09–4.09). High PA was associated with a significantly lower risk of poor glycaemic control compared with low PA (OR 0.20, 95% CI 0.05–0.68). In combined analyses, the greatest reduction in the risk of poor glycaemic control was observed in participants with good sleep quality and moderate PA, compared to those with poor sleep quality and low PA (OR 0.38, 95% CI 0.14–0.98).

**Conclusion:**

In patients with T2DM, achieving optimal glycaemic control requires not only maintaining PA but also improving sleep quality.

## 1 Introduction

Given the increasing prevalence of type 2 diabetes mellitus (T2DM) globally and the significant burden it imposes, identifying modifiable risk factors for poor glycaemic control is crucial to prevent complications (1). Adequate glycaemic control plays a pivotal role in slowing the progression of T2DM and reducing its associated complications (2, 3). Poor glycaemic control can lead to hyperglycaemic symptoms and immediate complications (4). However, a recent study indicated that only half of patients with diabetes achieve adequate glycaemic control (5), suggesting that inadequate control is becoming more prevalent.

Among the various factors influencing glycaemic control, sleep has emerged as an important candidate (6, 7). Sleep disturbances are common both in the general population and among patients with T2DM, and their prevalence is rising due to the demands of modern society (1, 8, 9). Previous observational studies have shown that both sleep duration and quality are associated with hemoglobin A1c (HbA1c) levels in adults. For instance, the Fukuoka Diabetes Registry's cross-sectional study found a significant link between short or prolonged sleep and elevated HbA1c levels (10).

Physical activity (PA) is another key factor that has demonstrated effectiveness in improving clinical outcomes, including mortality, comorbidities, and HbA1c levels in T2DM patients (11–13). Regular PA is considered a core component of glycaemic management in diabetes, with moderate to vigorous physical activity (MVPA) shown to reduce HbA1c in a dose-dependent manner (14). Furthermore, MVPA enhances muscle glucose uptake and long-term glycaemic control by increasing skeletal muscle oxidative capacity and improving insulin signaling (15).

Sleep and PA are interrelated health behaviors (16, 17). Sleep and PA are closely related in terms of energy expenditure and time utilization (18, 19). Both acute and regular PA have been shown to improve sleep quality, while the quality and duration of sleep also affect PA performance (20–22). Previous studies have examined the separate effects of sleep quality and PA on glycaemic control in T2DM patients, yet inconsistencies in the measures used and the findings reported remain. Therefore, this study aims to explore both the independent and joint associations of sleep quality and PA with glycaemic control in community-based patients with T2DM, providing evidence-based guidance for improving glycaemic control through sleep and PA interventions.

## 2 Methods

### 2.1 Data source and study sample

Tis cross-sectional study used data from the community disease profiles of the Provincial Chronic Disease Prevention and Control Demonstration Area programme, conducted between May and July, 2019, in Bengbu City, China. All patients with T2DM—diagnosed in hospital before the survey—within the jurisdiction of a community health service station were recruited using whole-population sampling. The study protocol was approved by the Ethics Committee of Bengbu Medical College [Approval No. (2016) 15], and written informed consent was obtained from all participants.

Eligible participants were adults (≥18 years) with T2DM who had resided in the study area for more than 6 months during the preceding 12 months. Exclusion criteria were: (1) history of psychiatric or cognitive disorders; (2) severe organic disease or mobility impairment; (3) pregnancy or lactation; and (4) missing HbA1c data. A total of 329 patients with T2DM were included in the final analysis.

### 2.2 Evaluation of glycaemic control

Glycaemic control was defined according to the comprehensive control targets for type 2 diabetes mellitus outlined in the Chinese Guidelines for the Prevention and Control of Type 2 Diabetes Mellitus (2020 Edition), developed by the Diabetes Branch of the Chinese Medical Association. Achievement of glycaemic control was defined as a HbA1c level of <7.0% (23).

### 2.3 Sleep quality assessment

Sleep quality over the past month was assessed using the Pittsburgh Sleep Quality Index (PSQI). The PSQI consists of 19 items grouped into seven components: subjective sleep quality, sleep latency, sleep duration, habitual sleep efficiency, sleep disturbances, use of sleep medication, and daytime dysfunction. Each component is scored from 0 to 3, yielding a total score of 0–21, with higher scores indicating poorer sleep quality. A total PSQI score ≥5 was considered indicative of poor sleep quality (24–26).

### 2.4 PA levels assessment

PA levels were assessed using the Chinese version of the International Physical Activity Questionnaire (IPAQ) (26), and were expressed in terms of Metabolic Equivalents (METs). Participants were categorized into three groups based on the World Health Organization (WHO) guidelines on PA for adults, which have been operationalized in previous studies using IPAQ data as follows: low PA (<600 MET-min/week), moderate PA (600–1,200 MET-min/week), and high PA (≥1,200 MET-min/week) (17, 27).

### 2.5 Covariates

Based on prior research, potential baseline covariates included age, sex, income, smoking status, alcohol consumption, body-mass index (BMI), family history of diabetes, diabetes duration, medication use, fasting plasma glucose (FPG), and triglycerides (TG). Sociodemographic information (age, sex, monthly income, education level, and family history of diabetes), lifestyle behaviors (smoking and alcohol consumption), and diabetes-related factors (duration of diabetes and medication use) were obtained through structured questionnaires administered via centralized and household surveys. Anthropometric measurements—including systolic blood pressure (SBP), diastolic blood pressure (DBP), weight, height, and BMI—were collected by trained researchers using standardized procedures and calibrated instruments. BMI was calculated as weight (kg)/height (m^2^). For laboratory testing, participants fasted from 8:00 pm the night before the examination, resulting in a total fasting duration of at least 10 h prior to venous blood sample collection the following morning. Venous blood samples were collected the following morning to assess HbA1c, FPG, TG, and total cholesterol (TC), all expressed in mmol/L. Community physicians provided reminders about fasting requirements and verified the timing of the last meal before blood collection. Samples were transported to the affiliated hospital laboratory for analysis.

### 2.6 Statistical analysis

Normality tests were performed on continuous variables, revealing that all exhibited non-normal distributions. Non-normally distributed continuous variables were reported as medians with interquartile ranges, and intergroup differences were assessed using the Wilcoxon signed-rank test. Categorical variables were presented as frequencies with proportions, and intergroup differences were evaluated using the chi-square test. Multivariate logistic regression analyses were conducted to explore the associations between sleep quality, PA, and glycaemic control in patients with T2DM. Adjustments were as follows: Model 1 was unadjusted; Model 2 was adjusted for age, sex, income, smoking, and alcohol consumption; and Model 3 was further adjusted for additional potential confounders, including BMI, family history of diabetes, duration of diabetes, medication use, FPG, and TG. Joint analyses were performed based on combined strata of sleep quality (good/poor) and PA (low/moderate/high), with the poor sleep quality and low PA group serving as the reference. In addition, a multiplicative interaction between sleep quality and PA was formally tested by including their product term in the logistic regression model. Complementing these primary analyses, we also examined the associations by treating PSQI scores and log-MET as continuous exposures, assessing potential non-linear relationships.

Finally, two sensitivity analyses were conducted to assess the robustness of the findings. First, categorical variables for age and BMI were used for adjustment. Second, additional adjustments were made for carbohydrate, fat, protein, and total energy intake to determine whether diet influenced the relationship between sleep quality/PA and glycaemic control. Multiple imputation was applied to minimize the reduction in sample size due to missing covariates. All statistical tests were conducted at a significance level of 0.05. Data analyses were performed using R version 4.40 and MSTATA software (https://www.mstata.com/).

## 3 Results

### 3.1 Participant characteristics

A total of 329 participants (45.0% male, 55.0% female) were included in the study, comprising 142 individuals with well-controlled blood glucose and 187 with poorly controlled blood glucose. Baseline characteristics of the study population are presented in Table 1. Significant differences were observed between the groups in terms of income, BMI, family history of diabetes, duration of diabetes, medication use, FPG, and HbA1c levels (*p* < 0.05). Compared to the poorly controlled group, the well-controlled group had lower BMI, FPG, and HbA1c levels. Within the well-controlled group, a smaller proportion of participants had an income ≥4,000 CN¥ (70.4%), engaged in low PA (33.8%), had a family history of diabetes (33.1%), had a diabetes duration of ≥20 years (9.9%), or were using diabetes medication (64.8%). No significant differences were found between the groups in terms of age, gender, education level, smoking status, alcohol consumption, hypertension, TG, or TC levels (*p* > 0.05).

**Table 1:** Baseline characteristics of participants with T2DM.

**Category**	**Characteristic**	**Overall *N* = 329**	**Controlled diabetes *N* = 142**	**Uncontrolled diabetes *N* = 187**	***p*-value**
Demographic characteristics	**Age (years)**	66.0 (61.0, 71.0)	66.0 (60.0, 70.0)	66.0 (61.0, 71.0)	0.5
	**Gender**				0.5
	Male	148 (45.0)	61 (43.0)	87 (46.5)	
	Female	181 (55.0)	81 (57.0)	100 (53.5)	
	**Education level**				0.2
	<High School	217 (66.0)	88 (62.0)	129 (69.0)	
	≥High School	112 (34.0)	54 (38.0)	58 (31.0)	
	**Income (CN**¥**)**				**<0.001**
	<2,000	37 (11.2)	26 (18.3)	11 (5.9)	
	2,000–3,999	22 (6.7)	16 (11.3)	6 (3.2)	
	≥4,000	270 (82.1)	100 (70.4)	170 (90.9)	
Lifestyle behaviors	**Smoking status**				0.8
	Yes	84 (25.5)	37 (26.1)	47 (25.1)	
	No	245 (74.5)	105 (73.9)	140 (74.9)	
	**Alcohol consumption**				>0.9
	Yes	87 (26.4)	38 (26.8)	49 (26.2)	
	No	242 (73.6)	104 (73.2)	138 (73.8)	
	**PA**				**0.009**
	Low	129 (39.2)	48 (33.8)	81 (43.3)	
	Moderate	169 (51.4)	73 (51.4)	96 (51.3)	
	High	31 (9.4)	21 (14.8)	10 (5.3)	
Anthropometric measurements	**BMI (Kg/m** ^**2**^ **)**	24.7 (22.6, 27.3)	24.0 (22.3, 26.6)	25.2 (23.0, 27.8)	**0.015**
	**Hypertension**				>0.9
	Yes	181 (55.0)	78 (54.9)	103 (55.1)	
	No	148 (45.0)	64 (45.1)	84 (44.9)	
Diabetes-related factors	**Family history of diabetes**				**0.011**
	Yes	135 (41.0)	47 (33.1)	88 (47.1)	
	No	194 (59.0)	95 (66.9)	99 (52.9)	
	**Duration of diabetes (years)**				**<0.001**
	<5	100 (30.4)	60 (42.3)	40 (21.4)	
	5–9	73 (22.2)	30 (21.1)	43 (23.0)	
	10–14	71 (21.6)	26 (18.3)	45 (24.1)	
	15–19	43 (13.1)	14 (9.9)	29 (15.5)	
	≥20	42 (12.8)	12 (8.5)	30 (16.0)	
	**Medication use**				**<0.001**
	Yes	245 (74.5)	92 (64.8)	153 (81.8)	
	No	84 (25.5)	50 (35.2)	34 (18.2)	
Laboratory indicators	**FPG (mmol/L)**	7.8 (6.8, 9.9)	6.8 (6.2, 7.5)	9.3 (7.8, 10.9)	**<0.001**
	**HbA1c (%)**	7.1 (6.5, 8.2)	6.4 (6.0, 6.7)	8.1 (7.4, 9.1)	**<0.001**
	**TG (mmol/L)**	1.6 (1.1, 2.4)	1.5 (1.0, 2.2)	1.7 (1.1, 2.5)	0.086
	**TC (mmol/L)**	4.7 (4.0, 5.4)	4.6 (3.8, 5.3)	4.7 (4.1, 5.4)	0.3

Median (Q1, Q3); *n* (%). Wilcoxon rank sum test; Pearson's Chi-squared test.

PA, physical activity; HbA1c, hemoglobin A1c; BMI, body mass index; FPG, fasting plasma glucose; TG, triglycerides; TC, total cholesterol.

Bold values indicate statistical significance at *p* < 0.05.

### 3.2 Association between sleep quality and glycaemic control

Table 2 presents the association between sleep quality and glycaemic control in patients with T2DM. In the multivariate logistic regression model (Model 3), habitual sleep efficiency was significantly associated with glycaemic control (OR = 1.64, 95% CI: 1.14–2.43), indicating that a decline in habitual sleep efficiency was associated with an increased risk of suboptimal glycaemic control. In Models 1 and 2, sleep disturbance emerged as a risk factor for poor glycaemic control (OR = 1.94, 95% CI: 1.30–2.95; OR = 1.82, 95% CI: 1.20–2.81), with higher PSQI scores reflecting a higher risk of suboptimal glycaemic control (OR = 1.10, 95% CI: 1.02–1.19; OR = 1.10, 95% CI: 1.02–1.20). However, this association was not statistically significant in Model 3. Additionally, participants with poor sleep quality had a higher risk of suboptimal glycaemic control compared to those with good sleep quality (OR = 2.09, 95% CI: 1.09–4.09). When treated as a continuous variable, the PSQI score was not significantly associated with glycaemic control, although a non-linear relationship was suggested (*p* for non-linearity = 0.038; see Supplementary Table 1).

**Table 2:** Logistic regression analysis of sleep quality and glycaemic control.

**Characteristic**	**Model 1**		**Model 2**		**Model 3**	
	**OR (95% CI)**	***p***	**OR (95% CI)**	***p***	**OR (95% CI)**	***p***
Subjective sleep quality	0.94 (0.69, 1.28)	0.711	0.92 (0.66, 1.27)	0.605	1.14 (0.74, 1.78)	0.553
Sleep latency	1.10 (0.90, 1.35)	0.339	1.09 (0.88, 1.35)	0.449	1.06 (0.79, 1.41)	0.711
Sleep duration	1.27 (0.99, 1.64)	0.062	1.22 (0.94, 1.59)	0.137	1.20 (0.84, 1.72)	0.320
Habitual sleep efficiency	1.48 (1.13, 1.98)	**0.005**	1.50 (1.13, 2.03)	**0.007**	1.64 (1.14, 2.43)	**0.007**
Sleep disturbances	1.94 (1.30, 2.95)	**0.001**	1.82 (1.20, 2.81)	**0.005**	1.26 (0.71, 2.26)	0.434
Sleep medications	1.11 (0.80, 1.58)	0.531	1.23 (0.86, 1.79)	0.261	1.10 (0.70, 1.75)	0.691
Daytime dysfunction	1.11 (0.88, 1.40)	0.393	1.14 (0.89, 1.46)	0.306	0.99 (0.70, 1.39)	0.941
PSQI score	1.10 (1.02, 1.19)	**0.011**	1.10 (1.02, 1.20)	**0.015**	1.09 (0.98, 1.21)	0.122
Sleep quality		**0.002**		**0.001**		**0.029**
Good	—		—		—	
Poor	2.07 (1.31, 3.29)		2.25 (1.39, 3.67)		2.09 (1.09, 4.09)	

OR, odds ratio; CI, confidence interval; PSQI, Pittsburgh sleep quality index.

Model 1: no covariates were adjusted;

Model 2: adjusted for age, gender, income, smoking status and alcohol consumption;

Model 3: further adjusted for BMI, family history of diabetes, duration of diabetes, medication use, FPG and TG.

Bold values indicate statistical significance at *p* < 0.05.

### 3.3 Association between PA and glycaemic control

Multivariate logistic regression analysis was conducted to examine the association between PA and glycaemic control. In the unadjusted model, the risk of suboptimal glycaemic control was significantly lower in the high PA group compared to the low PA group (OR = 0.28, 95% CI: 0.12–0.64). After adjusting for all covariates, the risk of suboptimal glycaemic control remained significantly lower in the high PA group (OR = 0.20, 95% CI: 0.05–0.68). The moderate PA group did not show a statistically significant association in any of the models. The results are presented in Table 3. The median weekly PA volume for each category is provided in Supplementary Table 2. The analysis of log-transformed MET as a continuous variable confirmed a significant protective effect against poor glycaemic control (OR: 0.77, 95% CI: 0.59–0.97), with evidence of a non-linear dose-response relationship (*p* for non-linearity = 0.021; Supplementary Table 1).

**Table 3:** Logistic regression analysis of PA and glycaemic control.

**Characteristic**	**Model 1**		**Model 2**		**Model 3**	
	**OR (95% CI)**	***p***	**OR (95% CI)**	***p***	**OR (95% CI)**	***p***
**PA**						
Low	–		–		–	
Moderate	0.78 (0.49–1.24)	0.298	0.69 (0.42–1.13)	0.144	0.73 (0.37–1.39)	0.335
High	0.28 (0.12–0.64)	**0.003**	0.26 (0.11–0.61)	**0.002**	0.20 (0.05–0.68)	**0.012**

OR, odds ratio; CI, confidence interval; PA, physical activity.

Model 1: no covariates were adjusted;

Model 2: adjusted for age, gender, income, smoking status and alcohol consumption;

Model 3: further adjusted for BMI, family history of diabetes, duration of diabetes, medication use, FPG and TG.

Bold values indicate statistical significance at *p* < 0.05.

### 3.4 Joint effect of sleep quality and PA on glycaemic control

The results of the joint effects analysis are summarized in Table 4. In Model 3, the risk of suboptimal glycaemic control was most significantly reduced in the group with good sleep quality and moderate PA compared to the group with poor sleep quality and low PA (OR = 0.38, 95% CI: 0.14–0.98). The risk of suboptimal glycaemic control was also significantly lower in the good sleep quality and high PA group compared to the poor sleep quality and low PA group (OR = 0.15, 95% CI: 0.03–0.72). No statistically significant differences were observed in the remaining groups. Additionally, the interaction between sleep quality and PA on glycaemic control was not significant (see Supplementary Table 3). Finally, Supplementary Table 4 displays the unadjusted HbA1c levels for all combinations of sleep quality and PA.

**Table 4:** Analysis of the joint effect of sleep quality and PA on glycaemic control.

**Joint group**	**Model 1**	**Model 2**	**Model 3**
	**OR (95% CI)**	**OR (95% CI)**	**OR (95% CI)**
Poor sleep quality & Low PA	—	—	—
Good sleep quality & Low PA	0.84 (0.39–1.83)	0.82 (0.36–1.88)	0.74 (0.25–2.19)
Poor sleep quality & Moderate PA	1.04 (0.58–1.86)	0.93 (0.50–1.72)	0.90 (0.41–1.98)
Good sleep quality & Moderate PA	**0.40 (0.20–0.78)**	**0.33 (0.16–0.68)**	**0.38 (0.14–0.98)**
Poor sleep quality & High PA	0.40 (0.11–1.36)	0.41 (0.11–1.48)	0.26 (0.04–1.39)
Good sleep quality & High PA	**0.20 (0.06–0.58)**	**0.18 (0.05–0.53)**	**0.15 (0.03–0.72)**

OR, odds ratio; CI, confidence interval; PA, physical activity.

Model 1: no covariates were adjusted;

Model 2: adjusted for age, gender, income, smoking status and alcohol consumption;

Model 3: further adjusted for BMI, family history of diabetes, duration of diabetes, medication use, FPG and TG.

Bold values indicate statistical significance at *p* < 0.05.

### 3.5 Sensitivity analysis

In sensitivity analyses, we observed consistent results when adjusting for age (<60 and ≥60 years) and BMI (<28 and ≥28 kg/m^2^) by categorizing continuous variables (Model 4, Supplementary Table 5). Furthermore, in Model 5, based on Model 4, the associations between subjective sleep efficiency, sleep quality, PA, and glycaemic control remained largely unchanged after additional adjustment for protein, fat, carbohydrate, and total energy intake (Supplementary Table 6). Similar results were observed for interaction effects (Supplementary Tables 7, 8).

## 4 Discussion

Our findings suggest that in community-based patients with T2DM, decreased habitual sleep efficiency and poorer sleep quality are associated with an increased risk of suboptimal glycaemic control. Compared with individuals with low PA, those engaging in high PA exhibited a significantly lower risk of poor glycaemic control. Additionally, the combined analysis revealed that individuals with good sleep quality and moderate PA experienced the greatest reduction in the risk of suboptimal glycaemic control.

Several prior studies have explored the relationship between sleep and glycaemic control in T2DM patients. For instance, poorer subjective sleep quality (10) and reduced sleep efficiency (28–30) have been linked to higher HbA1c levels in individuals with T2DM. Our results are consistent with a recent study in Mexico, which found that poor sleep quality doubled the risk of poor glycaemic control, even after accounting for other risk factors (31). Additionally, a cross-sectional study from Iran also highlighted a significant association between habitual sleep efficiency and poor glycaemic control, aligning with our findings (32).

The American Diabetes Association's Standards of Care have recently incorporated sleep health, reflecting its growing relevance to glycemic management (33, 34). Our study strengthens this emerging evidence by demonstrating a clear link between poor sleep quality and suboptimal glycemic control in a community-based T2DM population. From a pathophysiological perspective, our observations are consistent with mechanisms highlighted in experimental research, including disruptions in sleep architecture (e.g., suppressed slow-wave sleep) and consequent sympathetic nervous system activation, which are known to impair insulin secretion and glucose metabolism (35–37). While causal inferences cannot be drawn from our cross-sectional data, these findings collectively underscore the value of considering sleep quality in clinical practice. Further studies are needed to clarify these underlying mechanisms.

In addition to sleep, PA plays a critical role in improving glycaemic control in T2DM patients. Numerous meta-analyses have demonstrated the effectiveness of various PA regimens in managing glycaemic levels (38–41). However, no studies have definitively identified the optimal type or dose of PA needed to validate these recommendations. A recent study identified a non-linear dose-response relationship between PA and glycaemic control, showing that the optimal dose for T2DM patients was 1,100 MET-min per week, irrespective of baseline HbA1c levels (42).

The association between PA and improved HbA1c observed in our study is consistent with several established physiological mechanisms, such as increased glucose uptake in skeletal muscle (43), improved adipocyte function, and reduced cytokine production (44). Furthermore, evidence suggests that moderate-intensity endurance training can enhance glycaemic control and late-phase β-cell function (45), which may partly explain the beneficial association observed in the group with both good sleep quality and moderate PA. However, individual responses to PA are highly variable (46), and excessive PA can lead to mitochondrial damage and impaired glucose tolerance (47), which may influence the relationship between PA and HbA1c changes.

Our study also found that good sleep quality combined with moderate PA significantly reduced the risk of suboptimal glycaemic control. However, we did not observe a significant interaction between sleep quality and PA in relation to glycaemic control. Existing literature suggests a degree of interdependence between PA and sleep, with both influencing health through distinct mechanisms (16, 48–50). PA potentially influencing sleep through mechanisms such as fluctuations in body temperature, glucose metabolism, autonomic nervous system activity, and changes in body composition, mood, and cardiorespiratory health (21, 22, 51).

Moreover, the observed benefit of combining good sleep with moderate PA supports the notion that good sleep quality may help mitigate the risks associated with lower activity levels. This could be facilitated by mechanisms that promote metabolic homeostasis, such as those improving glucose regulation (35, 43). This observation helps explain our finding that while only high levels of PA reduced the risk of suboptimal glycaemic control, the combination of good sleep quality and moderate PA significantly lowered the risk. Future research should further explore the underlying mechanisms between sleep and PA through longitudinal studies, and investigate personalized exercise intervention strategies for individuals with sleep disorders.

## 5 Strengths and limitations

Our study has several strengths. First, it is the first to examine the joint association between sleep quality, PA, and glycaemic control, while also exploring potential interactions between these factors. Second, we carefully adjusted for a range of potential confounders, including sociodemographic factors, lifestyle behaviors, and diabetes-related variables such as duration and medication status. Lastly, we conducted sensitivity analyses, particularly excluding dietary factors, to assess the robustness of our findings.

However, this study has several limitations. First, it involves a relatively small sample, limiting the generalizability of the results. Moreover, the sample size may have provided insufficient statistical power to detect significant interaction effects between sleep quality and PA, even if such interactions truly exist. Second, the cross-sectional design precludes causal inference regarding the relationships between sleep quality, PA, and glycemic control. The observed associations may be subject to reverse causality, whereby poor glycemic control could lead to sleep disturbances and reduced PA, as well as residual confounding. Furthermore, several specific limitations in confounding control must be noted. Although we adjusted for the use of hypoglycemic medications, we could not account for specific drug classes (e.g., insulin, sulfonylureas, GLP-1 receptor agonists, SGLT2 inhibitors), which have distinct effects on glycemia and sleep. We also did not systematically assess or adjust for obstructive sleep apnea (OSA) or depressive symptoms, both of which are strongly associated with sleep, PA, and glycemic control (52). Finally, the reliance on self-reported questionnaires for both sleep quality and PA introduces the potential for recall and response biases. Additionally, the multiple statistical comparisons conducted for the various components of sleep quality should be interpreted with caution. Self-reported PA may not adequately capture absolute activity levels compared to device-based measures (53–55), and similarly, objective sleep metrics are often more accurate than self-reports (56–58). The use of objective or combined assessment tools in future studies is therefore strongly recommended.

## 6 Conclusion

In conclusion, this study found that in patients with T2DM, reduced habitual sleep efficiency and poorer sleep quality are associated with an increased risk of suboptimal glycaemic control. Additionally, individuals with high PA levels were at a significantly lower risk of suboptimal glycaemic control compared to those with low PA. The combination of good sleep quality and moderate PA was linked to the greatest reduction in the risk of poor glycaemic control. These findings illustrate the potential of a comprehensive approach to T2DM management that addresses both sleep quality and PA to achieve optimal glycemic control. Future prospective and intervention studies are warranted to confirm these observed associations and establish their temporal sequence.

## Data Availability

The raw data supporting the conclusions of this article will be made available by the authors, without undue reservation. Requests to access these datasets should be directed to Hong Xie, xh@bbmu.edu.cn.
