# Supplementary material for: Independent and joint associations of sleep quality and physical activity with glycaemic control in patients with type 2 diabetes mellitus
Source: Front Nutr. 2025 Nov 3;12:1694982. doi: 10.3389/fnut.2025.1694982 (PMC12620479; doi:10.3389/fnut.2025.1694982)
Supplement: Supplementary file 1 [file Table_1.docx]

**Supplementary Table 1**： Logistic Regression Results of Continuous and Nonlinear Effects of MET (log-transformed) and PSQI Score on Glycaemic Control

| **Characteristic** | **Continuous Effect (OR, 95% CI)** | **Nonlinear Effect (OR, 95% CI)** | **p-value (Continuous)** | **p-value (Nonlinear)** |
| --- | --- | --- | --- | --- |
| MET | 0.77 (0.59-0.97) | ns(MET, df=3)2: -4.23 (0.02) | **0.034** | **0.021** |
|  |  | ns(MET, df=3)3: -4.89 (0.04) |  | **0.044** |
| PSQI Score | 1.09 (0.98-1.21) | ns(PSQI Score, df=3)2: 5.75 (0.04) | **0.122** | **0.038** |
|  |  | ns(PSQI Score, df=3)1: 1.45 (0.10) |  | 0.103 |
| Abbreviations: OR = Odds Ratio, CI = Confidence Interval; PSQI: Pittsburgh Sleep Quality Index; MET, Metabolic Equivalent of Task (log-transformed). | | | | |

**Supplementary Table 2**： Physical Activity Volume by Category

| **PA Category** | **N** | **MET-min/week, Median (IQR)** |
| --- | --- | --- |
| Low | 129 | 280 (77-459) |
| Moderate | 169 | 868 (759-1014) |
| High | 31 | 1314 (1299-1400) |
| Abbreviations: PA, Physical Activity; MET, Metabolic Equivalent of Task; IQR, Interquartile Range. | | |

**Supplementary Table 3**: Interaction between sleep quality and PA on glycaemic control

| **Group** | **Model 1** | **Model 2** | **Model 3** |
| --- | --- | --- | --- |
|  | OR(95%CI) | OR(95%CI) | OR(95%CI) |
| Poor sleep quality × Low PA | — | — | — |
| Good sleep quality × Moderate PA | 0.45(0.16-1.24) | 0.43(0.15-1.24) | 0.56(0.13-2.36) |
| Good sleep quality × High PA | 0.59(0.10-3.32) | 0.53(0.08-3.18) | 0.81(0.07-9.82) |
| OR = Odds Ratio, CI = Confidence Interval; PA: Physical Activity.  Model 1: no covariates were adjusted;  Model 2: adjusted for age, gender, income, smoking status and alcohol consumption;  Model 3: further adjusted for BMI, family history of diabetes, duration of diabetes, medication use, FPG and TG. | | | |

**Supplementary Table 4**：Unadjusted HbA1c Levels by Joint Strata of Sleep Quality and PA

| **Joint Group** | **N** | **HbA1c (%), Median (IQR)** |
| --- | --- | --- |
| Poor sleep quality & Low PA | 89 | 7.40 (6.60-8.70) |
| Good sleep quality & Low PA | 40 | 7.25 (6.25-7.93) |
| Poor sleep quality & Moderate PA | 111 | 7.30 (6.60-8.50) |
| Good sleep quality & Moderate PA | 58 | 6.90 (6.32-7.75**)** |
| Poor sleep quality & High PA | 12 | 6.60 (6.20-8.22) |
| Good sleep quality & High PA | 19 | 6.60 (6.15-7.60) |
| Abbreviations: PA, Physical Activity; IQR, Interquartile Range. | | |

**Supplementary Table 5**: Logistic regression analysis of sleep quality and PA in relation to glycaemic control, after converting age and BMI to categorical variables

| **Characteristic** | **Model 4** | | |
| --- | --- | --- | --- |
|  | OR | 95% CI | p-value |
| **Habitual sleep efficiency** | 1.63 | 1.14, 2.40 | **0.010** |
| **Sleep quality** |  |  | **0.026** |
| Good | — | — |  |
| Poor | 2.12 | 1.10, 4.14 |  |
| **PA** |  |  |  |
| Low | — | — |  |
| Moderate | 0.72 | 0.38, 1.38 | 0.323 |
| High | 0.19 | 0.05, 0.63 | **0.009** |
| OR = Odds Ratio, CI = Confidence Interval; PA: Physical Activity.  Model 4: Adjusted for age group(<60, ≥60), gender, income, smoking status and alcohol consumption, BMI group(<28, ≥28), family history of diabetes, duration of diabetes, medication use, FPG and TG. | | | |

**Supplementary Table 6:** Logistic regression analysis of sleep quality and PA in relation to glycaemic control, following further adjustment for dietary factors

| **Characteristic** | **Model 5** | | |
| --- | --- | --- | --- |
|  | OR | 95% CI | p-value |
| **Habitual sleep efficiency** | 1.61 | 1.12, 2.37 | **0.013** |
| **Sleep quality** |  |  | **0.019** |
| Good | — | — |  |
| Poor | 2.24 | 1.15, 4.45 |  |
| **PA** |  |  |  |
| Low | — | — |  |
| Moderate | 0.73 | 0.38, 1.39 | 0.338 |
| High | 0.18 | 0.05, 0.61 | **0.008** |
| OR = Odds Ratio, CI = Confidence Interval; PA: Physical Activity.  Model 5: Adjusted for age group(<60, ≥60), gender, income, smoking status and alcohol consumption, BMI group(<28, ≥28), family history of diabetes, duration of diabetes, medication use, FPG, TG, protein, fat, carbohydrate and energy. | | | |

**Supplementary Table 7:** Analysis of the joint effect of sleep quality and PA on glycaemic control, after converting age and BMI to categorical variables

| **Joint Group** | **Model 4** | | |
| --- | --- | --- | --- |
|  | OR | 95% CI | p-value |
| Poor sleep quality & Low PA | — | — |  |
| Good sleep quality & Low PA | 0.76 | 0.25, 2.25 | 0.616 |
| Poor sleep quality & Moderate PA | 0.89 | 0.41, 1.95 | 0.779 |
| Good sleep quality & Moderate PA | 0.38 | 0.14, 0.98 | **0.049** |
| Poor sleep quality & High PA | 0.25 | 0.04, 1.40 | 0.125 |
| Good sleep quality & High PA | 0.14 | 0.02, 0.64 | **0.017** |
| OR = Odds Ratio, CI = Confidence Interval; PA: Physical Activity.  Model 4: Adjusted for age group(<60, ≥60), gender, income, smoking status and alcohol consumption, BMI group(<28, ≥28), family history of diabetes, duration of diabetes, medication use, FPG and TG. | | | |

**Supplementary Table 8:** Analysis of the joint effect of sleep quality and PA on glycaemic control, following further adjustment for dietary factors

| **Joint Group** | **Model 5** | | |
| --- | --- | --- | --- |
|  | OR | 95% CI | p-value |
| Poor sleep quality & Low PA | — | — |  |
| Good sleep quality & Low PA | 0.72 | 0.23, 2.19 | 0.561 |
| Poor sleep quality & Moderate PA | 0.89 | 0.40, 1.95 | 0.770 |
| Good sleep quality & Moderate PA | 0.35 | 0.13, 0.92 | **0.037** |
| Poor sleep quality & High PA | 0.21 | 0.03, 1.27 | 0.094 |
| Good sleep quality & High PA | 0.12 | 0.02, 0.60 | **0.015** |
| OR = Odds Ratio, CI = Confidence Interval; PA: Physical Activity.  Model 5: Adjusted for age group(<60, ≥60), gender, income, smoking status and alcohol consumption, BMI group(<28, ≥28), family history of diabetes, duration of diabetes, medication use, FPG, TG, protein, fat, carbohydrate and energy. | | | |
